# Dementia Risk factors Disparities and Population‐Attributable Fraction in Ethiopia

**DOI:** 10.1002/alz70860_097005

**Published:** 2025-12-23

**Authors:** Muluken Azage Yenesew

**Affiliations:** ^1^ University of California San Francisco, San Francisco, CA, USA

## Abstract

**Background:**

Globally, fourteen modifiable risk factors (RFs) account for 45% of dementia cases. Addressing these risk factors collectively can potentially reduce the prevalence of dementia. However, limited data exist on such factors in Africa including Ethiopia. Thus, the study aimed to estimate the population‐attributable fractions (PAFs) for the 14 RFs in Ethiopia, assessing dementia risk factors by region and sex.

**Method:**

This study used a cross‐sectional analysis of the 14 RFs from the Ethiopian WHO STEP survey, which included 9,800 people. The prevalence of risk factors and communalities were derived from national WHO step survey data. Relative risks from the Lance 2024 meta‐analysis report were used to calculate the weighted PAFs.

**Result:**

The overall PAF was 51.4% (95% CI = 50.9–51.8%). The largest PAFs were less education = 15.4% (95% CI 15.0‐15.8), hearing loss=8.7% (95%CI 8.3‐9.1), social isolation = 5.7 (95% CI 5.3‐6.1), depression = 3.6 (95%CI 3.2‐4.0), and traumatic brain injury=3.6 (95%CI 3.2‐4.0). Smoking and heavy alcohol consumption adversely affected men more significantly than women, but lower educational attainment and obesity had a more pronounced effect on women than men. High LDL cholesterol, diabetes, hypertension, and obesity exerted a more significant influence on urban residents, but lower educational attainment and smoking had a higher effect on rural inhabitants.

**Conclusion:**

The study estimated a higher PAF for dementia in Ethiopia than the world population. Dementia modifiable risk factors varied by sex and place of residence. Interventions should be developed by addressing the unique risk factors associated with sex and place of residence in reducing the burden of dementia in Ethiopia.

